# Identification of the Phosphorus-Solubilizing Bacteria Strain JP233 and Its Effects on Soil Phosphorus Leaching Loss and Crop Growth

**DOI:** 10.3389/fmicb.2022.892533

**Published:** 2022-04-29

**Authors:** Haiyang Yu, Xiaoqing Wu, Guangzhi Zhang, Fangyuan Zhou, Paul R. Harvey, Leilei Wang, Susu Fan, Xueying Xie, Feng Li, Hongzi Zhou, Xiaoyan Zhao, Xinjian Zhang

**Affiliations:** ^1^Shandong Provincial Key Laboratory of Applied Microbiology, Ecology Institute, Qilu University of Technology (Shandong Academy of Sciences), Ji’nan, China; ^2^CSIRO Agriculture and Food, Glen Osmond, SA, Australia

**Keywords:** *Pseudomonas* sp., P-solubilizing bacteria (PSB), 2-keto gluconic acid (2KGA), soil P leaching test, plant growth promotion

## Abstract

Phosphorus (P) is one of the most limiting nutrients in global agricultural ecosystems, and phosphorus-solubilizing bacteria (PSB) can convert insoluble P into soluble P, thereby improving the absorption and use of soil P by plants. Increasing leaching loss of soil P due to PSB that could lead to water eutrophication is a major concern, although no direct experimental evidence is available to evaluate these effects. In this study, a highly efficient PSB strain, *Pseudomonas* sp. JP233, was isolated from soil and its P-solubilizing agent was identified by metabolomics and HPLC analyses. The effects of JP233 on P contents in soil leachates were also analyzed by microcosm leaching experiments in the absence and presence of maize. JP233 could solubilize insoluble P into soluble forms, and the molybdate reactive phosphorus (MRP) content reached 258.07 mg/L in NBRIP medium containing 5 g/L Ca_3_(PO_4_)_2_ within 48 h. Metabolomics analysis demonstrated that the organic acid involved in JP233 P solubilization was primarily 2-keto gluconic acid (2KGA). Further, HPLC analysis revealed that 2KGA contents rapidly accumulated to 19.33 mg/mL within 48 h. Microcosm leaching experiments showed that MRP and total phosphorus (TP) contents in soil leaching solutions were not significantly higher after JP233 inoculation. However, inoculation with JP233 into maize plant soils significantly decreased MRP and TP contents in the soil leaching solutions on days 14 (*P* < 0.01), 21 (*P* < 0.01), and 28 (*P* < 0.05). Inoculation with strain JP233 also significantly increased the biomass of maize aerial components and that of whole plants (*P* < 0.05). Thus, strain JP233 exhibited a significant plant-growth-promoting effect on maize development. In conclusion, the application of PSB into soils does not significantly increase P leachate loss. Rather, the application of PSB can help reduce P leachate loss, while significantly promoting plant absorption and use of soil P.

## Introduction

Phosphorus (P) is an essential nutrient for plants that is involved in diverse biochemical processes including lipid metabolism and the biosynthesis of nucleic acids and cell membranes (Ha and Tran, 2014). However, P is one of the most limiting nutrients in global agricultural ecosystems (Lin et al., 2016). P that is applied to soils can accumulate in non-labile forms due to its high-affinity chemical reactions and its occlusion to soil minerals and organic matter, leading to the prevalence of “legacy P.” The accumulation of legacy P in soils and potential transfer to water bodies ultimately leads to environmental concerns of eutrophication (Gatiboni et al., 2020). Therefore, the development of methods to improve the bioavailability of immobilized P in agricultural soils are critical (Yu et al., 2019) to mitigate the continued application of P beyond that which required by plants.

The use of phosphorus-solubilizing bacteria (PSB) could represent promising management strategies for improving P use efficiency. PSB are capable of converting insoluble P into soluble forms that are bioavailable for plant uptake (Sharma et al., 2013; Paulucci et al., 2015; Khan et al., 2021). Isolates have been obtained from numerous environments including agricultural soils, rhizosphere soils, sewage sludges, with *Pseudomonas* and *Bacillus* species most represented among cultures (Sharma et al., 2013; Yu et al., 2019; Chawngthu et al., 2020; Kaur and Kaur, 2020). The addition of PSB to agricultural settings have led to growth enhancement effects on maize, wheat, soybean, barley, sesame, century plant, and wild mint (Afzal et al., 2010; Pereira and Castro, 2014; Bautista-Cruz et al., 2019; Prakash and Arora, 2019; Chouyia et al., 2020; Kusale et al., 2021; Nithyapriya et al., 2021). Further, previous studies have shown that PSB can reduce soil legacy P by increasing use efficiency from fertilizers and improving long-term efficiency of agricultural production (Gatiboni et al., 2020).

The reported mechanisms of P solubilization by PSB in soils involve the release of complexes or mineral dissolving compounds (e.g., organic acid anions and extracellular enzymes) into the environment, or alternatively, through the direct release of P during substrate degradation. Organic acids are the basis of inorganic P solubilization in PSB *via* their lowering of soil pH, enhancing chelation of cations bound to P, competing with P for adsorption sites on soils, and forming soluble complexes with metal ions that are associated with insoluble P (Sharma et al., 2013).

Phosphorus-solubilizing bacteria are capable of converting insoluble P into soluble forms, although concerns have been raised regarding increased leaching-loss of soil P by PSB. However, PSB can help improve plant absorption and use of soil P, thereby helping reduce leaching-loss of P. Nevertheless, the association between P solubilizing effects of PSB and risks of P leaching-loss has not been studied. The aims of the present study were to: (1) isolate highly efficient PSB from agricultural ecosystems, (2) identify mechanisms of P solubilization by the PSB, (3) investigate the direct influence of PSB on P leaching without plants, and (4) analyze the influence of PSB on P leaching with plants, in addition to assessing their influence on plant development. This study provides a foundation for informing PSB application to agricultural ecosystems.

## Materials and Methods

### Isolation of Phosphorus-Solubilizing Bacteria From Agriculture Soils

Soil samples were collected from the vegetable greenhouse facility of the Research and Development Base at the Shouguang Facility Agriculture Center, Chinese Academy of Sciences (118°86′82″ E, 36°90′99″ N). Soil physicochemical properties included a pH = 7.81, available *N* = 84 mg/kg, available *P* = 109.85 mg/kg, available *K* = 224.97 mg/kg, total *P* = 2,754.87 mg/kg, total *K* = 5,639.67 mg/kg, and organic matter content = 1.93%. The soil particle size distribution included 8.39% clay (<2 μm), 69.89% silt (2–50 μm), and 21.72% sand (50–2,000 μm).

About 10 g of soil samples were mixed with 90 mL of sterilized ddH_2_O (dd’H_2_O) and homogenized by shaking for 30 min in a conical flask. After serial dilution (10^–2^–10^–6^) with dd’H_2_O, a 100 μL aliquot of each suspension was plated on National Botanical Research Institute’s phosphate growth medium (NBRIP) agar plates [including per liter: 10 g glucose, 5.0 g MgCl_2_.6H_2_O, 0.25 g MgSO_4_.7H_2_O, 0.1 g (NH_4_)_2_SO_4_, 0.2 g KCl, 5.0 g Ca_3_(PO_4_)_2_, and 15 g agar, with pH adjusted to 7.5–8.0] (Nautiyal, 1999). Cultures were incubated at 28°C for 7 days. Bacterial colonies were subsequently selected from plates based on the appearance of a clear halo. Bacterial colonies, which can produce a clear halo zone on a plate, indicate the production of organic acids into the surrounding medium. The isolated strains were purified over five times, and each isolate following purification was stored in a glycerol stock at −20°C.

### Determination of Phosphorus Contents in Soils and Soil Leachates

Molybdate reactive phosphorus (MRP) was measured to directly analyze the P contents of samples using the molybdenum-antimony colorimetric method and by filtering soil leachate with a 0.45 μm microporous filter membrane (Heckrath et al., 1995). Total phosphorus (TP) was determined by adding 4 mL of 50 g/L potassium persulfate to leachate without filtration treatment, digestion at 120°C for 30 min in an autoclave sterilizer and measuring P concentrations of the digested P with molybdenum blue colorimetry (Huang, 2019).

Total phosphorus was determined using the potassium persulfate digestion method (Su and Chen, 2010). Soil samples were first air-dried, ground, and sieved, followed by mixing with 5 mL of hot potassium persulfate solution. The mixture was heated in an autoclave sterilizer until the pressure reached 1.1 kg/cm and the temperature was 120°C, where it was maintained for 30 min. The solution was then cooled to room temperature, transferred to a 100 mL volumetric flask to a constant volume, and filtered with phosphorus-free filter paper. An aliquot (2 mL) of the filtered sample was pipetted into a 50 mL volumetric flask, two drops of a dinitrophenol indicator were added, and the pH was adjusted with sodium carbonate solution and sulfuric acid solution until a yellowish color was observed. Subsequently, 5 mL of a molybdenum-antimony stock solution (13 g ammonium molybdate, 0.35 g antimony potassium tartrate, 150 mL concentrated sulfuric acid, and dd’H_2_O to 500 mL) was added along with 1 mL of a 10% ascorbic acid solution. The mixture was maintained at room temperature for 30 min, and the absorbance at a wavelength of 700 nm was determined. Available phosphorus (AP) was determined using the Olsen method (Bao, 2000). Briefly, samples were passed through a 2 mm sieve after air drying and sodium bicarbonate was added prior to oscillating filtration for 30 min. Then, 5 mL of filtrate was mixed with the molybdenum-antimony chromogenic agent, followed by addition of water up to 50 mL, maintenance at room temperature for 30 min, and measurement of absorbance at a 700 nm wavelength.

### Determination of Phosphorus Solubilizing Capacity for Selected Strains

Each isolated PSB colony was inoculated into NBRIP liquid medium and incubated on a rotary shaker at 180 r/min and 30°C for 48 h, followed by measurement of soluble P contents in each fermentation culture. To measure P content, a small amount of fermentation broth was centrifuged at 10,000 r/min for 10 min and 1 mL of supernatant was removed and diluted, if necessary. Then, 1 mL of molybdenum-antimony antibody solution and 0.5 mL of ascorbic acid solution was added in turn, mixed well, and reacted at room temperature for 15 min. The samples without P were used as blank controls. The MRP contents were then determined as described in section “Determination of P Contents in Soils and Soil Leachates.” Strains with strong P solubilizing ability were stored in glycerol in a −20°C freezer until later use.

### Amplification and Molecular Identification of 16S rRNA Genes of the Phosphorus-Solubilizing Bacteria Strain JP233

Strain JP233 exhibited relatively strong P solubilizing ability and was further identified using 16S rRNA gene sequencing. Genomic DNA of the strain was extracted (using Bacterial DNA Kit, OMEGA Bio-Tek, Suite 450400 Pinnacle Way, Norcross, GA, United States) and 16S rRNA genes of the strain were amplified by PCR using universal primers for bacteria: 27F (5′-AGAGTTTGATCCTGGCTCAG-3′) and 1492R (5′-GGGTTACCTTGTTACGACTTC-3′). PCR reaction program included pre-denaturation at 95°C for 5 min, followed by 35 cycles of denaturation at 94°C for 40 s, annealing at 52°C for 30 s and extension at 72°C for 90 s, and all followed by a final extension at 72°C for 10 min. The PCR amplification products were submitted to Sangon Biotech (Shanghai) Co., Ltd. (Shanghai, China) for DNA sequencing. The resulting 16S rRNA gene sequences were aligned and compared with references in the NCBI GenBank database.

### Metabolome Analysis

Treatment group samples were inoculated with the stable JP233 strain in NBRIP liquid medium, while the control group was NBRIP liquid medium without strain inoculation. Each group comprised six independent biological replicates. Cultivation was conducted at 150 r/min for 48 h at 28°C. A total of 1 mL of bacterial suspension was removed from each treatment, centrifuged at 3,000 r/min at 4°C for 10 min, and 800 μL of the supernatant was transferred to a cryovial and placed in a −80°C freezer for preservation. The samples were then subjected to non-targeted metabolomics analysis at LC-Bio Co., Ltd. (Hangzhou, China).

The samples were thawed on ice and metabolites were extracted from 20 μL of each sample using 120 μL of pre-cooled 50% methanol buffer, followed by vortexing for 1 min and incubating for 10 min at room temperature and then storage at −20°C overnight. The mixture was then centrifuged at 4,000 *g* for 20 min and the supernatant was subsequently transferred to 96 well plates. Pooled quality control (QC) samples were also prepared by combining 10 μL of each extraction mixture. All samples were analyzed using a TripleTOF 5600 Plus high-resolution tandem mass spectrometer (SCIEX, Warrington, United Kingdom) in both positive and negative ion modes. Chromatographic separation was performed with an ultra-performance liquid chromatography (UPLC) system (SCIEX, Warrington, United Kingdom). An ACQUITY UPLC T3 column (100 mm × 2.1 mm, 1.8 μm, Water, United Kingdom) was used for reversed-phase separation. The mobile phase consisted of solvent A (water, 0.1% formic acid) and solvent B (acetonitrile, 0.1% formic acid), while the column temperature was maintained at 35°C. The TripleTOF 5600 Plus system was used to detect metabolites eluted from the column. The ion spray floating voltage was set at 5 kV for the positive-ion mode and at −4.5 kV for the negative-ion mode. A QC sample was analyzed every 10 samples to evaluate LC-MS stability. The acquired LC-MS data pretreatment was conducted with the XCMS software program. Raw data files were converted into the mzXML format and processed using the XCMS, CAMERA, and metaX toolboxes for the R software environment. Each ion was identified using comprehensive retention time and m/z information and these data were matched to both in-house and public databases. The open access databases KEGG^1^ and HMDB^2^ were used to annotate metabolites by matching exact molecular mass data (m/z) to those from the databases, when considering a threshold of 10 ppm. Peak intensity data was further pre-processed using the metaX program. Features that were detected in <50% of the QC samples or <80% of the test samples were removed and values for missing peaks were extrapolated using the k-nearest neighbor algorithm to further improve data quality. The relative standard deviations of metabolic features were calculated across all QC samples and those with standard deviations >30% were removed. Data normalization was performed on all samples using the probabilistic quotient normalization algorithm. The QC-robust spline batch correction was then used for QC samples. *p* values were calculated with student *t*-tests that were adjusted for multiple tests using a false discovery rate (FDR) (Benjamini-Hochberg) for different metabolite selection. A variable importance for the projection (VIP) cut-off value of 1.0 was used to identify important features.

### Determination of 2-Keto Gluconic Acid Production Capacity of JP233

The 2-keto gluconic acid (2KGA) standard solution was prepared by precisely weighing 0.1000 g of the 2KGA standard and dissolving it with distilled water, followed by bringing it to a constant volume in a 50 mL volumetric flask to prepare a 2 mg/mL stock solution. The stock solution was diluted with distilled water to achieve 2KGA standard solutions with concentrations of 2 mg/mL, 1.5 mg/mL, 1 mg/mL, 0.8 mg/mL, 0.5 mg/mL, and 0.1 mg/mL, mixed well and then filtered with 0.22 μm aqueous microporous filter membranes. The solutions were then subjected to degassing treatments using an ultrasonic cleaner for 15 min and subsequent high-performance liquid chromatography (HPLC) determination (Wang et al., 2014).

Pretreatment of samples for testing included first inoculating strain JP233 into NBRIP liquid medium and culturing with shaking at 180 r/min for 6 days at 30°C. The fermentation broth was sampled every 12 h and filtered with a disposable sterile filter (0.22 μm pore diameter) and the filtrate was collected to directly determine the pH and 2KGA contents. The pH of the fermentation broth was measured with a pH meter and 2KGA contents were determined with HPLC (Niu et al., 2012). HPLC was used to characterize the samples using an Agilent 1200 system with an Aminex HPX-87H (300 mm × 7.8 mm, 9 μm) column, 5 mM H_2_SO_4_ as the mobile phase, a column temperature of 55°C, a flow rate of 0.6 mL/min, a detection wavelength of 210 nm, and 20 μL sample sizes, while using a DAD detector. Peak areas were determined from chromatograms and standard curves were constructed with the peak areas as the ordinate and the concentrations of the standard working solution as the abscissa. The standard curve equation was *y* = 913.15x + 12.353 (*R*^2^ = 0.9998).

### Effects of Phosphorus-Solubilizing Bacteria Strain JP233 on Phosphorus Leaching From Soils

Microcosm experiments were designed to investigate the effects of JP233 on P leaching. Specifically, a PVC tube with an inner diameter of 7 cm and a length of 40 cm was placed in the soil column (as described in section “Isolation of Phosphorus-Solubilizing Bacteria From Agriculture Soils”) (Xie et al., 2019). Petrolatum was first evenly applied to the inner wall of the tube and a layer of 300 mesh nylon mesh was placed on the bottom of the column, followed by layering of about 60 g of quartz sand (about 1 cm thickness), and then a layer of 300 mesh nylon mesh that was placed to prevent soil infiltration. Finally, 1,800 g of test soil was loaded into the upper portion of the nylon mesh based on the actual soil bulk density in the field. KH_2_PO_4_ (0.44 g) was mixed into the surface soil and the loaded soil column was placed above a beaker to collect leachate. Three soil columns were established in the treatment and control groups, and 600 mL of deionized water was added to each column to achieve the maximum water holding capacity and ensure consistency of soil conditions in each column. Activated JP233 was inoculated into 250 mL of nutrient broth medium and placed in a shaker at 180 rpm and at 28°C for 24 h. The bacterial solutions were centrifuged twice and 150 mL of dd’H_2_O was used to resuspend cells, followed by application to the soil columns. In place of cultures, 150 mL of dd’H_2_O only was applied to soil columns in the control group. After inoculation, 100 mL of water was added to the soil columns every 3 days to replenish soil water, while leached fluid was collected into a beaker and TP and MRP contents in the leachate fluids were measured (as described in section “Determination of P Contents in Soils and Soil Leachates”) on the same day. The experiments lasted 30 days, and the total phosphorus loss was calculated from the measurements. The calculation formulae used were:


(1) $$\text{MRP}\,{\mathrm{mass}}_{n}=\mathrm{MRP}\,{\mathrm{concentration}}_{\text{n}}\times \mathrm{Leachate}\,{\mathrm{volumen}}_{\text{n}}$$

(2) $$\mathrm{Total}\,\mathrm{MRP}\,\mathrm{mass}=\sum_{i=1}^{\text{n}}{\text{MRPmass}}_{\text{i}}$$

(3) TPmass=nTPconcentration×nLeachatevolumen

(4) $$\mathrm{Total}\,\mathrm{TP}\,\mathrm{mass}=\sum_{i=1}^{n}{\mathrm{TPmass}}_{i}$$

where n indicates the nth watering. At the end of the experiment, soil samples were removed in layers (0–10 cm, 10–20 cm, 20–30 cm, and 30–40 cm layers), and the soil samples were air-dried and sieved to determine TP and AP mass concentrations in each layer of soil samples (as described in section “Determination of P Contents in Soils and Soil Leachates”).

### Effects of Phosphorus-Solubilizing Bacteria Strain JP233 on Soil Phosphorus Leaching and Maize Growth

Maize seeds with full grains were selected and pre-germinated on moist filter paper for 24 h before sowing. Two experimental groups were established in the experiment, with the treatment group being inoculated with JP233 cultures and the control group without bacterial inoculation. Three independent biological replicates were used for each group. The soil column leaching device used for the experiment was the same as described in section “Determination of 2-Keto Gluconic Acid Production Capacity of JP233.” Activated JP233 was inoculated into 3 mL of nutrient broth medium, individually inoculated into three tubes, and placed in a shaker for cultivation over 48 h. Bacterial solutions were then centrifuged twice (5,000 r/min), supernatants were discarded, and the bacterial solutions were resuspended with sterile water to an OD_600_ of 1.0. Six pre-germinated seeds per treatment were soaked in the resuspended bacterial solutions for 30 min, and two were sown in each treatment soil column after soaking. Six pre-germinated seeds per control were soaked in sterile water for 30 min and two were placed in each control soil column after soaking.

After sowing, each soil column was watered every 7 days over 28 days (i.e., at days 7, 14, 21, and 28) based on maize growth water requirements, with watering volumes of 150, 300, 550, and 400 mL, respectively. Soil leachates were collected after each watering to determine TP and MRP contents (as described in sections “Determination of P Contents in Soils and Soil Leachates” and “Effects of Phosphorus-Solubilizing Bacteria Strain JP233 on P Leaching From Soils”). At the end of the pot culture experiments, the soil columns were divided into two layers comprising the 0–20 cm and 20–40 cm layers, and their rhizosphere and non-rhizosphere soils were retrieved. The soil samples were air-dried and sieved to determine TP and AP mass concentrations (as described in section “Determination of P Contents in Soils and Soil Leachates”). At the end of the leaching experiment, the maize plants were removed intact and oven-dried to a constant weight at 80°C. The dry matter mass of the aerial components and roots of the maize were separately weighed.

### Statistical Analyses

All experimental data were collated using Microsoft Excel 2016 and plotted using SigmaPlot 12.5. One-way analysis of variance (one-way ANOVA) was performed using the SPSS 24.0 statistical software program with all test data and using a significance level threshold of 0.05 or 0.01.

## Results

### Isolation of an Efficient Phosphorus-Solubilizing Bacteria Strain, JP233

**FIGURE 1:**
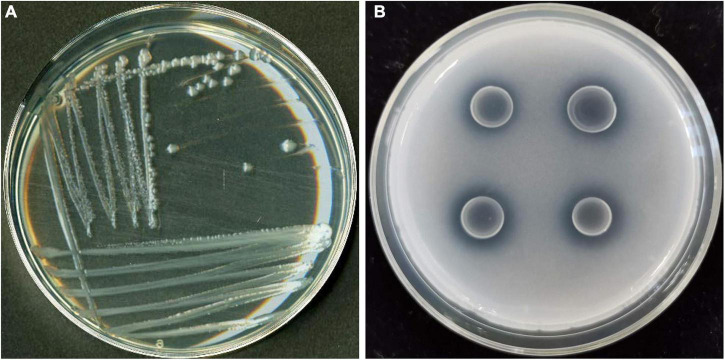
Colony morphology of PSB strain JP233 on an LB plate **(A)** and its characteristic P solubilizing halo on an NBRIP plate **(B)**.

Three PSB strains (identified as JP233, JP236, and J221) were screened based on the appearance of a clear halo on NBRIP agar plates. Soluble P content measurements in liquid culture demonstrated that strains JP233 (Figures 1A,B), JP236, and J221 released 258.07 ± 0.74, 110.11 ± 0.42, and 62.7 ± 0.35 mg/L soluble P from 5 g/L Ca_3_(PO_4_)_2_ within 48 h, respectively. Strain JP233 achieved the best P solubilization and was thus used for follow-up studies. 16S rRNA gene sequencing (GenBank ID: MW990045) analyses indicated that JP233 was highly related to some *Pseudomonas* species, such as *P. putida*, *P. plecoglossicida*, *P. moteilii*, *P. asiatica*, showing sequence identity bigger than 99.72%.

### Identification of the Critical Metabolite Involved in Phosphorus-Solubilization by JP233

**FIGURE 2:**
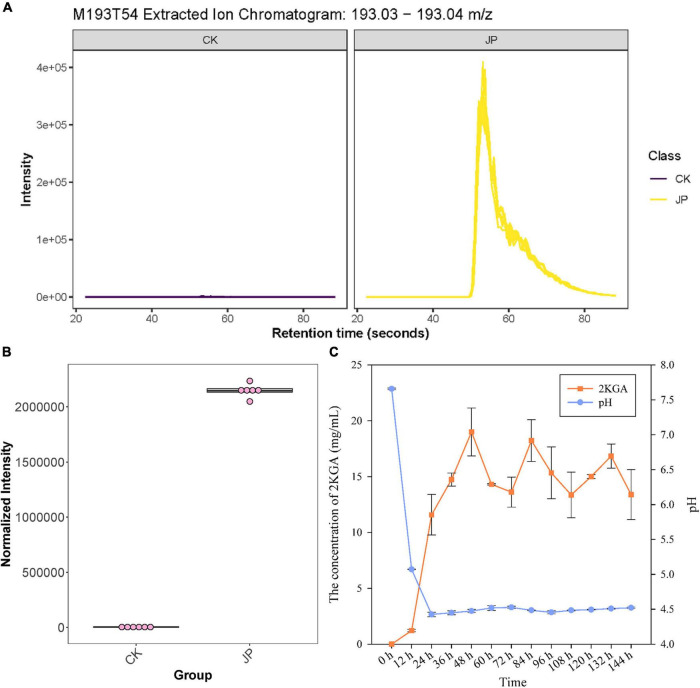
Metabolomic and HPLC identification and analysis of 2KGA. **(A)** The extracted ion chromatogram of compound M193T54 (identified as 2KGA *via* database alignment) from the JP233 treatment and CK. The yellow chromatogram indicates treatment and the purple one indicates CK. **(B)** Normalized intensity boxplot of M193T54 (2KGA) in the JP233 treatment and CK. **(C)** Changes of 2KGA concentration and the pH of JP233 fermentation broths. Results represent means ± SD (*n* = 6).

Untargeted metabolomics was used to evaluate the metabolites that differed between the JP233 treatment (JP) and the blank control (CK) groups cultured in NBRIP liquid medium for 48 h. Differential metabolites were identified as significantly different based on the ratio (JP/CK) >2, a *q* value (Benjamini-Hochberg’s adjustment) <0.05, and a VIP > 1. A total of 660 and 618 significantly upregulated metabolites were identified in negative- and positive-ion mode, respectively. Annotation of the metabolites was conducted using in-house and public databases (included KEGG and HMDB databases). Eleven upregulated metabolites were classified as organic acids including 2KGA (ratio = 567.60, *q* value = 1.87E-05, VIP = 3.17), (2-methoxyethoxy)propanoic acid (ratio = 1158.55, *q* value = 1.16E-07, VIP = 3.34), 2-oxopentanedioic acid (ratio = 977.61, *q* value = 1.75E-06, VIP = 3.31), D-(+)-pantothenic acid (ratio = 536.42, *q* value = 6.15E-07, VIP = 3.17), N-acetyl-L-glutamic acid (ratio = 38.17, *q* value = 2.86E-05, VIP = 2.46), *trans*-aconitic acid (ratio = 26.38, *q* value = 4.45E-06, VIP = 2.35), succinic acid (ratio = 23.05, *q* value = 2.43E-10, VIP = 2.25), 3-oxopentanoic acid (ratio = 8.45, *q* value = 1.09E-09, VIP = 1.87), maleic acid (ratio = 4.66, *q* value = 1.83E-09, VIP = 1.61), pantothenic acid (ratio = 106.80, *q* value = 3.19E-12, VIP = 2.83), and 2-ketohexanoic acid (ratio = 8.74, *q* value = 4.36E-07, VIP = 1.93). Moreover, the relative peak areas of the above organic acids exhibited large differences, with the largest being 2KGA, which was an order of magnitude larger than the organic acid with the second largest normalized intensity (Figures 2A,B).

High-performance liquid chromatography analysis was conducted to validate 2KGA production by strain JP233 in NBRIP liquid medium. 2KGA could be detected in the fermentation liquor of JP233 and concentrations rapidly rose within 48 h (Figure 2C), followed by fluctuations in concentrations in the range of 13.62–19.33 mg/mL until 156 h. Over the same experimental period, the fermentation liquor pH significantly dropped from an initial value of 7.66 to 4.43 within 24 h, followed by stabilization in the range of 4.43–4.53 until 156 h. The above results consequently indicated that JP233 could produce considerable concentrations of 2KGA in NBRIP medium and acidification occurred when 2KGA was produced. The preliminary conclusion was drawn that acidification caused by 2KGA production played an important role in P solubilization by JP233. It is noteworthy that a drastic drop in pH is observed within the first 12 h while production of 2KGA was afterward, which means the other organic acids also played roles in early acidification.

### Influence on Phosphorus Leaching by Direct Application of JP233 to Soils

**FIGURE 3:**
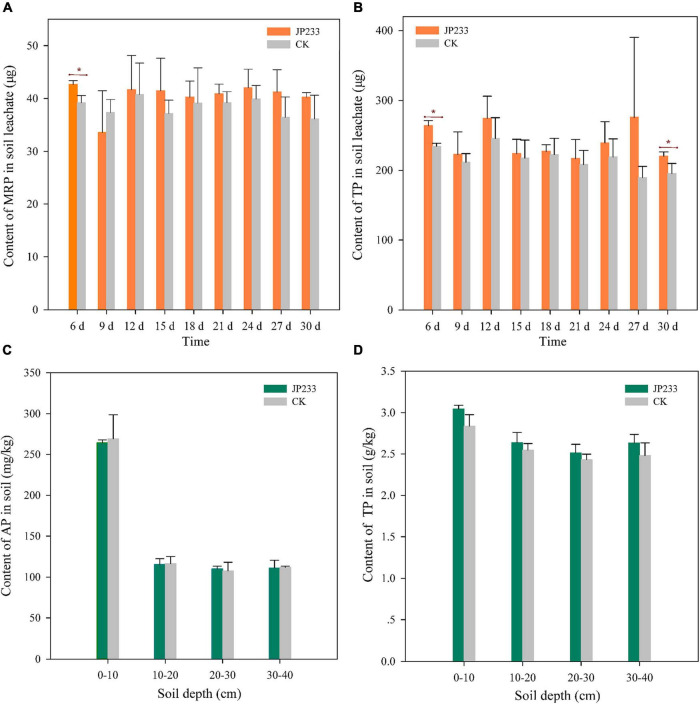
P contents in soil leachates and soils after JP233 treatment. **(A,B)** Show differences in MRP and TP contents in soil leachate. **(C,D)** Show differences of AP and TP contents in the soil column. Results represent means ± SD. One-way ANOVA was performed for each time or soil depth. *Statistically significant at *P* < 0.05.

Microcosmic experiments were conducted to analyze the influence of JP233 on P leaching from soils. MRP and TP levels were determined from soil leachates every 3 days from the 6th day until the 30th day in both treatment and control groups. On the first measurement time point (6 days post inoculation, dpi), MRP (*P* = 0.017, α = 0.05) and TP (*P* = 0.003, α = 0.01) levels in soil leachates were significantly higher in the JP233 treatment group than in the CK group. However, significant differences in MRP or TP loss between the treatment and the CK groups were not observed in subsequent leachate measurements, with the sole exception of TP loss (*P* = 0.049, α = 0.05) at 30 dpi (Figures 3A,B). After the leaching tests, the cumulative leaching loss of MRP and TP contents in soil leachates were calculated. The amounts of MRP and TP lost in the treatment group were 0.363 ± 0.027 mg and 2.16 ± 0.220 mg, respectively, while those in the control group were 0.345 ± 0.028 mg and 1.940 ± 0.130 mg, respectively. Thus, significant differences in total leaching loss of MRP (*P* = 0.448, α = 0.05) or TP (*P* = 0.209, α = 0.05) were not observed between the treatment and control groups.

The concentrations of TP and AP in the 0–10, 10–20, 20–30, and 30–40 cm soil layers were also measured after leaching experiments. AP and TP concentrations in the 0–10 cm soil layer were the highest, while P levels in the other soil layers were relatively similar. Further, statistically significant differences in the concentrations of AP or TP between the treatment and control groups were not apparent for each soil layer (Figures 3C,D).

The above results indicated that insoluble phosphorus was converted into soluble phosphorus after the PSB strain JP233 was applied to the soils, resulting in increased leaching of MRP and TP, although these differences were not statistically significant. Thus, the MRP and TP contents retained in the soils were essentially not different from those in the control.

### Influence on Phosphorus Leaching by Inoculation of JP233 Cultures to Maize Plant Soils

**FIGURE 4:**
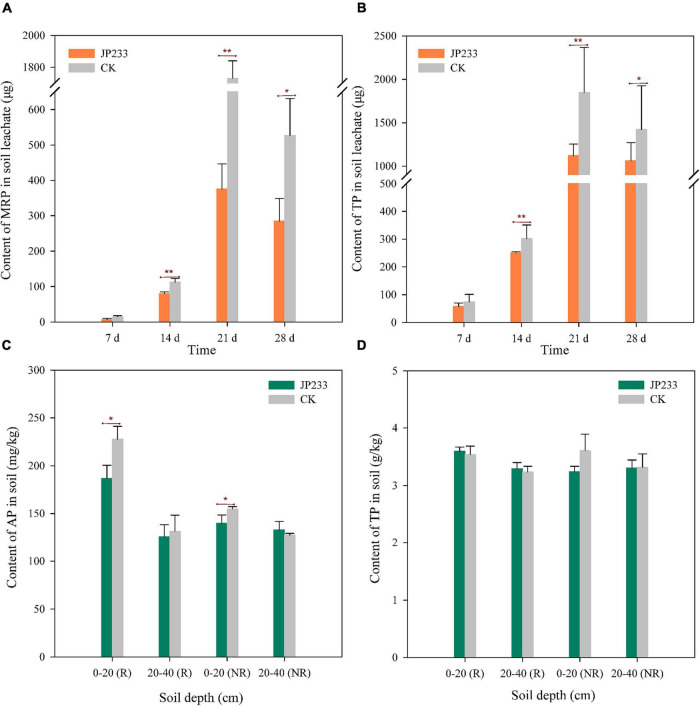
P contents in soil leachates and soils after JP233 treatment in maize plant soils. **(A,B)** Show differences in MRP and TP contents in soil leachate. **(C,D)** Show differences of AP and TP contents in the soil column. Results represent means ± SD. One-way ANOVA was performed for each time or soil depth. *Statistically significant at *P* < 0.05, **statistically significant *P* < 0.01.

Maize was planted in the same microcosm leaching devices described above and watered every 7 days according to maize seedling water requirements, followed by collecting leaching solution after each watering to measure MRP and TP. The treatment group was inoculated with JP233, while the control group was not inoculated with cultures. The MRP content of the treatment group was lower than that of the control group in each leaching test, with no significant difference in levels on day 7, but statistically significant differences on days 14 (*P* = 0.008, α = 0.01), 21 (*P* = 0.000, α = 0.01), and 28 (*P* = 0.026, α = 0.05) (Figure 4A). TP differences exhibited similar trends, with lower TP content in the treatment group than in the control group and without significant differences in levels on day 7, but statistically significant differences on days 14 (*P* = 0.000, α = 0.01), 21 (*P* = 0.002, α = 0.01), and 28 (*P* = 0.056, α = 0.05) (Figure 4B). After leaching tests, cumulative leaching loss of MRP and TP in soil leachates were calculated. MRP and TP concentrations in the treatment group were 0.747 ± 0.139 mg and 2.489 ± 0.232 mg, respectively, while these values in the control group were 2.384 ± 0.214 mg and 4.290 ± 0257 mg, respectively. Significant differences were observed in both total leaching loss of MRP (*P* = 0.000, α = 0.01) and TP (*P* = 0.001, α = 0.01) when comparing the treatment and control groups.

The mass concentrations of residual AP and total TP in rhizosphere and non-rhizosphere soils were determined after the leaching experiments. AP contents in the rhizosphere and non-rhizosphere soils at a depth of 0–20 cm in the treatment group were significantly lower than in the control group and significant differences were not observed in AP contents when compared between rhizosphere and non-rhizosphere soils at a depth of 20–40 cm between the two groups (Figure 4C). However, significant differences in TP content were not observed between rhizosphere and non-rhizosphere soils at both depths when comparing the two groups (Figure 4D).

**FIGURE 5:**
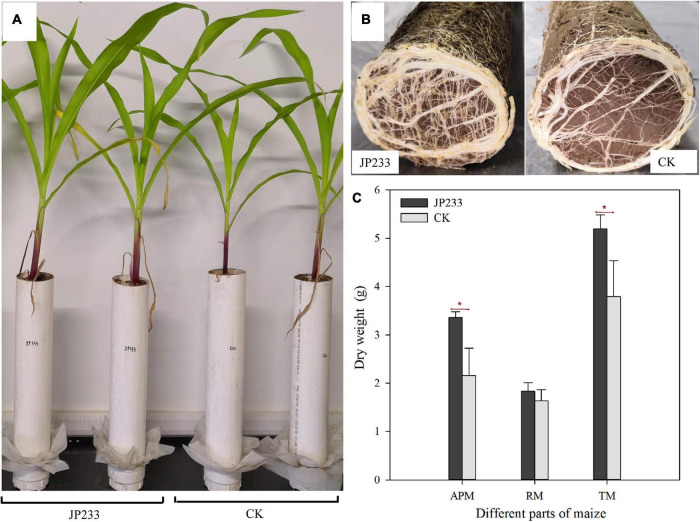
Effects of JP233 treatment on maize seedling biomass. **(A)** Aerial plant component growth status of JP233 treatment and CK group plants. **(B)** Root growth status at the bottom of the soil column of the JP233 treatment and CK group plants. **(C)** Differences in masses of JP233 treatment and CK dry matter. APM, aerial part mass; RM, root mass; TM, total mass. Results represent means ± SD. One-way ANOVA was performed for each time or soil depth. *Statistically significant at *P* < 0.05.

Maize biomass accumulation was also evaluated after the leaching experiments. JP233 inoculation significantly increased the dry matter mass of aerial maize components (*P* = 0.023, α = 0.05) (Figures 5A,C) and also increased the mass of root dry matter, although not significantly (Figures 5B,C), but did significantly increase the dry matter of the entire plant (*P* = 0.037, α = 0.05) (Figure 5C).

The above results indicated that MRP and TP contents of soil leachate after inoculation with strain JP233 into maize plant soils were significantly lower than in controls without inoculation. AP content retained in inoculated soils decreased compared to controls, while TP contents in soils were not different from controls. In addition, JP233 inoculation significantly increased dry matter quality and promoted maize growth.

## Discussion

Phosphorus-solubilizing bacteria are commonly used plant probiotics that promote plant development by converting insoluble P into soluble P that is easily absorbed and used by roots (Hamid et al., 2021). The mechanism of P solubilization by PSB is relatively clear and occurs primarily through the secretion of organic acids that dissolve inorganic phosphorus or *via* the secretion of phosphatases that degrade organophosphorus. P solubilizing bacteria are an important type of microbial fertilizer and many efficient strains have been developed into agents for application (Sarkar et al., 2021). However, it has remained unclear whether the solubilization of soil P by PSB will result in additional phosphorus migration into groundwater, thereby contributing to eutrophication of water bodies.

In this study, a highly efficient PSB strain, *Pseudomonas* sp. JP233, was isolated from agricultural soils that could release 258.07 mg/L MRP from 5 g/L Ca_3_(PO_4_)_2_ within 48 h, representing a P conversion efficiency of 25.81%. *Pseudomonas* strains have been demonstrated to vary in their ability to dissolve P, for example with the PSB strains *Pseudomonas* sp. P34, *P. fluorescens* MS-01, *P. putida* PSE3, *Pseudomonas* sp. RT5RP2, *Pseudomonas* sp. RT6RP, *P. lurida* M2RH3, and *P. putida* PSRB6 dissolving 101.60, 29.80, 319.00, 38.33, 35.40, 340.00, and 100.00 mg/L of soluble P, respectively (Hariprasad and Niranjana, 2009; Selvakumar et al., 2011, 2013; Ahmad et al., 2013; Kadmiri et al., 2018; Liu et al., 2019).

Metabolome analysis and HPLC determination identified 2KGA as the primary P solubilizing organic acid that is secreted by JP233. 2KGA is converted from gluconic acid in the periplasm by an FAD-dependent gluconate dehydrogenase that is encoded by the gad operon (Yum et al., 1997; Toyama et al., 2007; Saichana et al., 2009). Indeed, expression of the gad operon of *P. putida* KT 2440 in *Enterobacter asburiae* PSI3 improves mineral phosphate solubilization (Kumar et al., 2013). In addition, *Gluconobacter*, *Pseudogluconobacter*, *Pseudomonas*, *Serratia*, *Klebsiella*, and *Enterobacter* spp. have all been shown to produce 2KGA (Hwangbo et al., 2003; Li et al., 2016). 2KGA-producing *Pseudomonas* species include *P. corrugate*, *P. fluorescens*, *P. plecoglossicida*, and *P. aureofaciens* (Trivedi and Sa, 2008; Sun et al., 2013; Umezawa et al., 2015; Wang et al., 2019).

Other organic acids were also produced by JP233 including (2-methoxyethoxy) propanoic acid, 2-oxopentanedioic acid, D-(+)-pantothenic acid, N-acetyl-L-glutamic acid, *trans*-aconitic acid, succinic acid, 3-oxopentanoic acid, maleic acid, pantothenic acid, and 2-ketohexanoic acid. Acids released by PSB that have been shown to be involved in P solubilization include citric acid, formic acid, gluconic acid, 2-keto gluconic acid, lactic acid, malic acid, oxalic acid, propionic acid, and succinic acid (Khan et al., 2007; Sharma et al., 2013; Alori et al., 2017; Chakdar et al., 2018; Su et al., 2019; Monroy Miguel et al., 2020; Spohn et al., 2020). It should be noted that organic acids produced by strains of the genus are not identical. For example, the organic acids produced by *P. poae* BIHB 751 are gluconic acid, ketogluconic acid, citric acid and malic acid, while those by *P. trivialis* BIHB 769 are gluconic acid, ketogluconic acid, lactic acid, fumaric acid, malic acid, and succinic acid, and those by *Pseudomonas* sp. AZ15 are oxalic acid, gluconic acid, acetic acid, lactic acid, and citric acid (Vyas and Gulati, 2009; Zaheer et al., 2019; Rawat et al., 2021). Comparison against other strains revealed that strain JP233 exhibited a high capacity for P solubilization and produced a wide variety of organic acids.

The MRP and TP contents in soil leachate increased (albeit overall insignificantly) in microcosm leaching experiments when JP233 was applied to soils, although a significant increase was observed in the first leaching measurement at 7 days. The MRP and TP contents in surface soil layers were higher than those in deeper layers after 30 days, although statistically significant differences were not observed between the JP233 treatment and control soils. Thus, the application of PSB strain JP233 to soil could not alone improve the solubility of soil P, although a non-significant increase in P leaching loss was observed. This result could be due to the existence of abundant P-fixing compounds in the soils, in addition to the P-solubilizing effects of PSB being relatively limited. These results are also consistent with those previously reported (Yu et al., 2019), wherein strain PSB *P. prosekii* YLYP6 could produce 716 mg/L of AP under optimized conditions. This study also only observed a significant difference between treatment and control groups on the 10th day when measuring at days 4, 10, and 30.

When PSB JP233 was inoculated into maize plant soils, leaching loss of P significantly decreased. After watering on days 14, 21, and 28, the concentrations of MRP and TP lost to leakage in the treatment group were significantly lower than in the control group. Further, the amount of MRP and TP lost to leakage in the treatment group was lower than in the control group on day 7, albeit not significantly. Moreover, AP remaining in the rhizosphere and non-rhizosphere soils were also significantly lower than in the control at 0–20 cm depth, but not significantly different at 20–40 cm depth, nor were TP contents significantly different among different soil zones. In the results of this current study, P solubilization by PSB JP233 led to less P loss from pot experiments with plants compared to controls. A previous study hypothesized that organic acids in root exudates (e.g., oxalic acid) were involved in the mobilization of P within the rhizosphere (Ström, 1997), which may explain why maize itself resulted in excessive P loss. Regarding why the effect was minimized when PSB JP233 was present, possible explanations could be that the activation of P by PSB produced an active state of P that is more readily absorbed, or that PSB promoted the uptake of P by plants through unknown mechanisms; these mechanisms require further investigation. Concomitantly, the aerial component and whole dry matter weight of maize plants treated with JP233 were significantly larger compared to non-treated ones. Thus, JP233 inoculation promoted maize plant growth. These results suggest that the growing plant roots can absorb more soluble P from soil into plant tissues, while organic acids like 2KGA produced by JP233 dissolve more soluble P in soils. These results can be explained by the absorption of P by maize roots, rather than leaving soluble P in soils or accumulating it within leachate, thereby leading to significantly reduced leachate loss of P and concomitant significant increases of maize plant biomass of maize.

## Conclusion

In this study, the roles of PSB were investigated from an environmental perspective, focusing on the growth promotion effects of PSB on plants, while also analyzing the risk of PSB application on the transfer of soil soluble P to groundwater. These data were used to provide a foundation for the rational use of PSB. An efficient PSB strain, *Pseudomonas* sp. JP233, was isolated and identified from soils. Metabolomics and HPLC analyses revealed that its primary P solubilizing organic acid was 2KGA. Microcosm leaching tests indicated that JP233 alone did not significantly increase leaching loss of MRP and TP. When inoculated into maize plant soils, JP233 significantly reduced MRP and TP loss compared to controls. Moreover, inoculation with JP233 significantly increased the biomass of maize aerial components and for the whole plant.

## Data Availability Statement

The raw data supporting the conclusions of this article will be made available by the authors, without undue reservation.

## Author Contributions

HY: methodology, investigation, writing-original draft, data curation, and formal analysis. XW: conceptualization, writing-original draft, visualization, data curation, and formal analysis. GZ: resources and investigation. FZ: software and resources. PH: conceptualization and writing-review and editing. LW: methodology. SF, XX, and FL: investigation. HZ and XiaZ: validation. XinZ: conceptualization, supervision, project administration, funding acquisition, and writing-review and editing. All authors contributed to the article and approved the submitted version.

## Conflict of Interest

The authors declare that the research was conducted in the absence of any commercial or financial relationships that could be construed as a potential conflict of interest.

## Publisher’s Note

All claims expressed in this article are solely those of the authors and do not necessarily represent those of their affiliated organizations, or those of the publisher, the editors and the reviewers. Any product that may be evaluated in this article, or claim that may be made by its manufacturer, is not guaranteed or endorsed by the publisher.

## Footnotes

1
https://www.kegg.jp/

2
https://hmdb.ca/
