# Supplementary material for: Identification of the Phosphorus-Solubilizing Bacteria Strain JP233 and Its Effects on Soil Phosphorus Leaching Loss and Crop Growth
Source: Front Microbiol. 2022 Apr 29;13:892533. doi: 10.3389/fmicb.2022.892533 (PMC9100411; doi:10.3389/fmicb.2022.892533)
Supplement: Supplementary file 1 [file Data_Sheet_1.PDF]

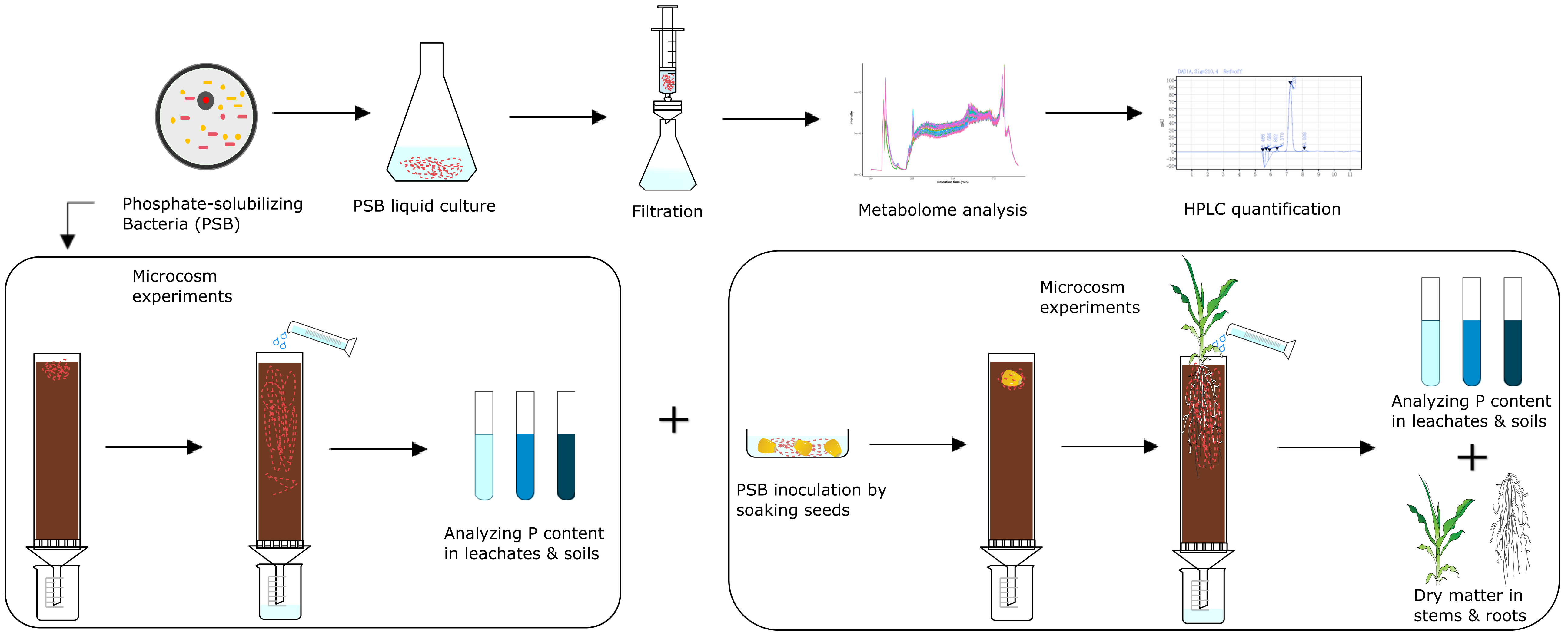
Phosphate-solubilizing Bacteria (PSB)
PSB liquid culture
Filtration
Metabolome analysis
HPLC quantification
Microcosm experiments
Analyzing P content in leachates & soils
+
PSB inoculation by soaking seeds
Microcosm experiments
Analyzing P content in leachates & soils
+
Dry matter in stems & roots
